# The factor structure and use of the PHQ-4 among high school learners in selected South African districts

**DOI:** 10.1192/j.eurpsy.2025.1275

**Published:** 2025-08-26

**Authors:** S. Mashegoane, J. O. August, V. Baloyi, L. D. M. Lebeloane, M. Makhubela, K. P. Moalusi

**Affiliations:** 1 Psychology, University of Limpopo, Polokwane; 2 Psychology, Nelson Mandeal University, Qheberha; 3 Psychology, University of Venda, Thohoyandou; 4 Further Teacher Education; 5 Industrial and Organisational Psychology, University of South Africa, Tshwane, South Africa

## Abstract

**Introduction:**

In this study, the scale’s psychometric properties are estimated among South African high school learners during a period of the coronavirus-19 crisis. The study was a cross-sectional design. The data were collected from 1603 high school learners in four South African districts. The PHQ-4 was administered for data collection, and was anchored from 0 (not at all) to 3 (nearly every day) over a two-week period, with higher scores indicating the extent of symptom severity (Kroenke et al., 2009).

**Objectives:**

The study sought to validate the PHQ-4 among South African school learners in various South African districts, and tested a model that included the PHQ-4 to investigate the obtaned factor structure’s usefulness.

**Methods:**

Useful questionnaires were collected from 1562 high school learners within a cross-sectional design. Grades 10 to 12 learners completed the PHQ-4 from March to May 2022.

**Results:**

Confirmatory Factor Analysis (CFA) was conducted to validate the factor structure of the scale. CFA results show that all PHQ-4 items load onto a single factor. The factor structure reported in this study is unique. Whilst the PHQ-4 is commonly considered to measure anxiety and depression, the results suggest that the scale measures a unidimensional, psychological distress factor among the learners. The total score of the PHQ-4, characterized as psychological distress, could be predicated by associated factors, although the coefficients obtained were statistically significant but weak. The PHQ-4 was predicted by the Perceived Vulnerability and the Germ Aversion subscales but not the Fear of COVID-19 scale in girls. But the PHQ-4 was predicted by the Fear of COVID-19 scale and not the Perceived Vulnerability and the Germ Aversion subscales among boys.

**Conclusions:**

Contrary to existing studies, the PHQ-4 did not consist of 2 factors. Evidence from the current study suggests that it should be used as a unidimensional, single factor scale. The PHQ-4 total scores of girls and boys were predicted by different factors, suggesting that gender should be considered an important factor when using the PHQ-4 in reseaerch.

**Disclosure of Interest:**

None Declared

